# Effects of different types of fibers and fiber mixes on fresh and hardened properties of ultra-high performance concrete

**DOI:** 10.1617/s11527-025-02875-8

**Published:** 2025-12-01

**Authors:** Jose Patiño, Luca Galli, Prannoy Suraneni

**Affiliations:** 1 EAFIT University, 050022 Medellín, Antioquia Colombia; 2 Civil and Architectural Engineering, University of Miami, Coral Gables, FL 33146 USA

**Keywords:** Ultra-high performance concrete, Fibers, Flexural strength, Toughness, Compressive strength

## Abstract

The effect of different types of fibers and fiber mixes on fresh and hardened properties of commercial ultra-high performance concrete (UHPC) were investigated. Six types of metallic and non-metallic commercially available fibers, including ones commonly used in UHPC and ones not investigated in prior work, were evaluated. Eighteen mixtures with different fiber dosages and combinations are prepared and tested for flowability, compressive strength, flexural properties, and bulk resistivity. Generally, the fibers, with the exception of polyethylene fibers did not affect flowability. All fibers affected mechanical behavior significantly. Depending on fiber type and dosage, compressive strength was − 23 to + 19% of the value of the mixture without fibers. Polyethylene fibers and their blends with steel fibers showed excellent flexural properties. Compared to the control mixture, selected binary mixtures showed improved flexural and compressive strength and synergies. Selected binary mixtures showed improved flexural strength and a lower cost per flexural strength, but a somewhat reduced compressive strength. Clearly, there are complex cost, compressive strength, flexural strength, and flow tradeoffs that can be leveraged based on the required application.

## Introduction

Ultra-high performance concrete, commonly known as UHPC or UHPFRC, is a cement-based composite material with an optimized particle size gradation that shows outstanding hardened properties compared to conventional concretes. Some of its most relevant characteristics are its high compressive strength (> 120 MPa), sustained post cracking tensile strength (> 5.2 MPa), and that it is virtually impervious to water, chlorides, and other deleterious agents [1]. These properties have positioned UHPC as a promising and versatile material in the construction industry, with a rapidly growing market, especially in infrastructure projects where rapid commissioning and high durability are crucial [2].

For UHPC to achieve high flexural and tensile strengths with ductile behavior, the usage of fiber reinforcement is required. The addition of high-carbon steel microfibers, commonly added in dosages between 2 and 3%, with a length (l_f_) of 13 mm and a diameter (d_f_) of 0.20 mm, has shown to be effective in improving flexural and tensile strengths, reducing cracking, and contributing to the ductile behavior of the material while maintaining fresh state properties, like self-consolidating behavior [3]. However, using high-carbon steel microfibers dramatically impacts UHPC costs, as they can represent 40–70% of the total cost [4–7]. In addition, exposed fibers on the surface of architectural elements tend to rust and degrade their appearance. Finally, as these fibers have a needle-like shape, they can cause injuries during handling. Therefore, to lower the cost of UHPC and prevent other unwanted effects associated with steel fibers, the use of alternative fibers and hybrid fiber mixes has become a topic of significant interest.

Several authors have studied the influence of different fiber combinations on the properties of cement composites and high-strength matrices, but relatively few have done such tests on UHPC. Some reported results indicate that fiber combinations may have synergistic effects that enhance the mechanical and durability performance of the material. The most relevant findings are summarized in Table 1.

**Table 1:** Summary of some previous studies on hybrid fiber systems

Authors	Fiber types	Hybrid systems	Relevant findings
[8]	SH, SS	SH-SS	Synergistic behavior was observed. The highest tensile capacity was achieved with a blend of 2% SH and 1% SS.
[9]	SH, SS, PV	SH-SS SH-PV	The inclusion of PV fibers had beneficial effects of microfibers in flexural performance.
[10]	SH, SS, ST	SH-SS SS-SS ST-SS	The addition of microfibers in hybrid systems was favorable for strain hardening and multiple cracking behavior. Improved tensile performance was achieved with the combination of ST-SS.
[11]	SH, SD, CE	SH-CE SD-CE	CE fibers did not have any considerable effect on mechanical properties, but when combined with steel fibers, a synergistic behavior was observed in flexural and shear performance.
[12]	SS, BA, PV, PE	SS-BA SS-PV SS-PE	SS-PE hybrid system showed a significant increase in tensile strength and strain capacity. BA had a negative effect on the compressive strength.
[13]	SS, PP, CA	SS-PP PP-CA	In general, combinations of macro and microfibers enhance the flexural properties of high-strength matrices.
[14]	SS, SH, PV	SS-SH SS-PV	Both SS-PV and SS-SH increased the flexural and tensile strengths. The hybrid systems also had a significant impact on the flexural toughness.

BA: Basalt; CA: Carbon; CE: Cellulose; PE: Polyethylene; PP: Polypropylene; PV: Polyvinyl Alcohol; SD: Steel Double Deformed; SH: Steel Hooked; SS: Steel Straight; ST: Steel Twisted

The reported findings show that fiber material, percentage addition, and fiber geometry (size, shape, and length-diameter ratio) influence the properties of UHPC. This study aims to investigate how six different fiber types, added in a proportion of 1 and 2% by volume, and their combinations, which have not been evaluated before, influence UHPC properties, identifying synergetic behaviors.

## Materials and methods

For this study, a proprietary UHPC mixture was used as the main matrix that served as a fixed variable, isolating the effect of the fiber type and dosage on the properties of interest.

### Commercial UHPC

The commercial UHPC is a preblended and prepacked dry UHPC mixture that, when mixed with the right proportion of water, high-range water-reducing (HRWR) admixture, and high-carbon steel microfibers, will meet the parameters described in Table 2. As with most UHPC materials, this mixture has a water-to-cementitious material ratio under 0.25 and uses a 2% addition of fiber by volume.

**Table 2:** Commercial UHPC critical properties (from the Technical Data Sheet)

Property	Value	Standard
Free Flow	200–260 mm	ASTM C1437 (using modifications described in ASTM C1856)
Compressive Strength at 28 days	150 MPa	ASTM C39 (using modifications described in ASTM C1856)
Flexural Strength at 28 days	18 MPa	ASTM C1609 (using modifications described in ASTM C1856)
Modulus of Elasticity	38 GPa	ASTM C469 (using modifications described in ASTM C1856)

### Fibers

**Fig. 1:**
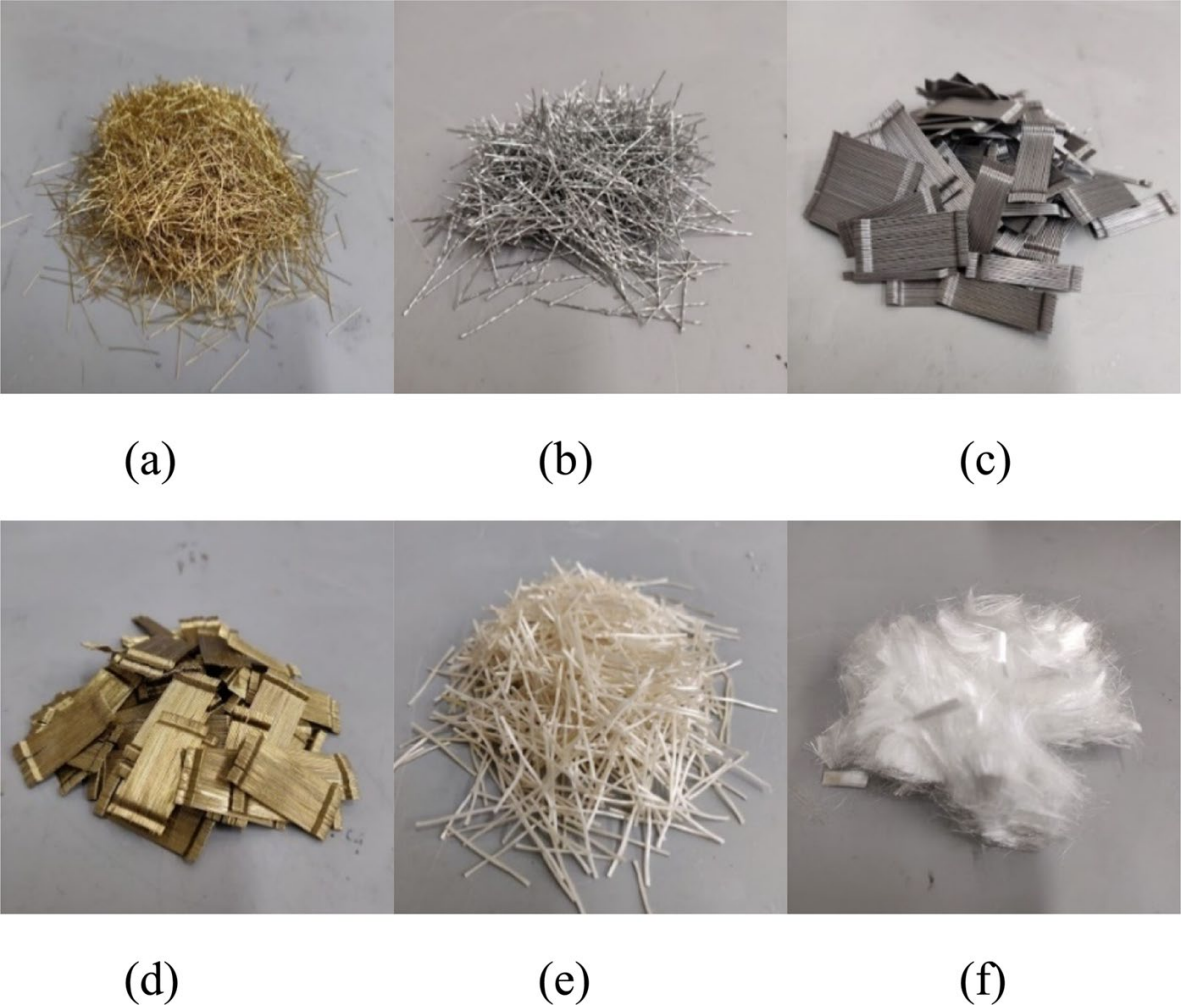
Fibers studied: **a)** CS; **b)** TS; **c)** HS; **d)** CH; **e)** GF; **f**) PE

Five commercially available and less-studied fibers were selected based on different aspects, such as cost, material, and geometry. The regularly used high-carbon steel fibers were also studied (CS). In the initial phase, four steel fibers: high-carbon (CS), twisted (TS), hooked (HS), and high-carbon hooked (CH), and two synthetic fibers: glass minibars (GF), and polyethylene fibers (PE), were evaluated. Fiber properties are described in Table 3, and an image of the fibers is shown in Fig. 1.

**Table 3:** Fiber types and properties

Name	Type of fiber	Diameter (µm)	Length (mm)	Aspect ratio (L/Ø)	Specific gravity	Tensile strength (MPa)	Modulus of elasticity (GPa)
CS	High carbon steel fiber	200	13	65.0	7.65	2750	200
TS	Twisted steel fiber	500	25	50.0	7.65	1700	200
HS	Hooked steel fiber	550	30	54.5	7.65	1345	200
CH	High carbon hooked steel fiber	380	30	78.9	7.65	3070	210
GF	Glass based fiber	700	24	34.3	2.00	1000	42
PE	Polyethylene fiber	15–20	12	800.0	0.97	2700	88

### Admixtures

UHPC has a very low water-to-cementitious materials ratio (around 0.20), so to achieve a flowable, self-consolidating mixture, it is necessary to use a HRWR admixture. In this case, a liquid polycarboxylate-based admixture with a solid mass content of 34% and a specific gravity of 1.08 was used at a dosage of 2.20% (with respect to the mass of the cementitious material).

### Test matrix

The experimental program consisted of Phase 1, where mixtures with each type of fiber were evaluated at dosages of 1 and 2% to characterize their individual performance. Based on Phase 1 results, Phase 2 was designed in which fiber mixtures were proposed and evaluated to determine their effect on the properties of interest in the UHPC and identify possible synergistic behaviors. Additionally, a baseline was established by evaluating a mixture without fibers (UHPC0). The mixture designs are summarized in Table 4. CS2, the mixture with 2% CS fibers is denoted as control, as that is the expected use case of the commercial UHPC.

**Table 4:** Mixture designs for the first and second phase

	Mixture designs (kg/m^3^)									
	Mixture	Argos UHPC	HRWR	Water	CS	TS	HS	CH	GF	PE
Phase 1	UHPC0	2043.1	22.1	236.3	**–**	**–**	**–**	**–**	**–**	**–**
	CS1	2043.1	22.1	236.3	76.5	–	–	–	–	–
	CS2 (Control)	2043.1	22.1	236.3	153.0	–	–	–	–	–
	TS1	2043.1	22.1	236.3	–	76.5	–	–	–	–
	TS2	2043.1	22.1	236.3	–	153.0	–	–	–	–
	HS1	2043.1	22.1	236.3	–	–	76.5	–	–	–
	HS2	2043.1	22.1	236.3	–	–	153.0	–	–	–
	CH1	2043.1	22.1	236.3	–	–	–	76.5	–	–
	CH2	2043.1	22.1	236.3	–	–	–	153.0	–	–
	GF1	2043.1	22.1	236.3	–	–	–	–	21.0	–
	GF2	2043.1	22.1	236.3	–	–	–	–	42.0	–
	PE1	2043.1	22.1	236.3	–	–	–	–	–	9.7
	PE2	2043.1	22.1	236.3	–	–	–	–	–	19.4
Phase 2	CS1HS1	2043.1	22.1	236.3	76.5	–	76.5	–	–	–
	CS1CH1	2043.1	22.1	236.3	76.5	–	–	76.5	–	–
	CS1GF1	2043.1	22.1	236.3	76.5	–	–	–	21.0	–
	CS1PE1	2043.1	22.1	236.3	76.5	–	–	–	–	9.7
	GF1.5PE0.5	2043.1	22.1	236.3	–	–	–	–	31.5	4.8

### Mixing and specimen preparation

Because of the low water content, a high shear mixer is needed to mix UHPC materials. In this case, a countertop 20 Qt planetary mixer with a flat agitator attachment was used. The mixing process was as follows. Approximately 70% of the dry mixture was loaded into the mixer bowl and mixed for 2 min at speed 1 (107 rpm). Then, 90% of the water and HRWR was added and mixed at speed 1 for 3 min. Further mixing was carried out at speed 2 (198 rpm) for 5 min until a flowable and homogenous consistency was achieved. At this time, the rest of the dry mixture and the remaining 10% water and HRWR was added and mixing was carried for 2 more minutes at speed 2. Finally, the fibers were added and mixing continued at speed 2 for 2 more minutes. Although UHPC is generally considered self-consolidating, all specimens were cast in a single layer and table-vibrated for 30 s at 60 Hz to remove entrapped air and ensure proper consolidation, particularly in mixes containing PE fibers, which prevent self-compacting behavior. External vibration was limited to avoid fiber segregation, especially for steel fibers with higher density than the matrix, in accordance with ASTM C1856, which prohibits internal vibration for this reason. After vibration, specimens were promptly wrapped with stretch plastic and stored at a controlled temperature of 23 ± 2 °C for 24 h before demolding. Curing was performed in a moist room compliant with ASTM C511 at standard conditions (23 ± 2 °C, free water on the surface) until testing. Post-test examination of specimens confirmed that no fiber segregation occurred.

### Test methods

Flow, compressive strength, and flexural performance tests were performed according to ASTM C1437, ASTM C39, and ASTM C1609, respectively, following the modifications described in ASTM C1856. Before testing, specimens were rotated 90° from their casting position to minimize the influence of casting direction on results. Cylindrical specimens (75 × 150 mm) were tested under compression using a servo-hydraulic testing machine (capacity 1780 kN) at a loading rate of 1.0 ± 0.05 MPa/s. Prismatic specimens (75 × 75 × 305 mm, span 254 mm) were tested under flexure using a third-point loading configuration. A universal electromechanical test system with a capacity of 133 kN, controlled by a data acquisition station, was used for flexural tests. Displacement was monitored and tests were performed at a net deflection rate of 0.075 mm/min until specimen failure, with displacement values up to 3.5 mm recorded for data significance. All specimens were tested at 28 days under laboratory conditions (23 ± 2 °C). As a comparative value, the apparent density of hardened UHPC mixtures was estimated by dividing the mass of 48-h air-dried specimens by their calculated volume. Bulk resistivity was measured in mixtures without steel fibers following ASTM C1876. Three specimens were tested for each proposed mixture.

## Results and discussion

Table 5 summarizes the results of fresh and hardened properties evaluated in the different UHPC mixtures. TS fibers produced an expansive effect on the UHPC specimens, leading to poor strengths. It was determined after discussion with the manufacturers that this expansion was due to a contaminating agent in their zinc coating. For this reason, the results related to TS fibers were not considered for further analysis.

**Table 5:** Mechanical and physical properties of different UHPC mixtures

Mixture	Average flow (mm)	f´c at 28 days (MPa)		σ at 28 days (MPa)*		Toughness T150 (J)	Bulk resistivity (Ωm)	Apparent density (g/cm^3^)	Theoretical density (g/cm^3^)
		Mean	SD	Mean	SD				
UHPC0	218	146.6	5.6	10.9	0.7	–	1035	2.30	2.30
CS1	208	168.6	7.2	14.5	0.3	28.3	–	2.39	2.38
CS2 (Control)	205	174.0	10.1	20.8	1.5	34.7	–	2.47	2.45
TS1**	218	119.5	4.2	9.3	1.0	14.3	–	2.35	2.38
TS2**	215	92.7	3.4	10.6	0.8	21.2	–	2.38	2.45
HS1	220	154.2	6.7	8.1	0.7	14.9	–	2.37	2.38
HS2	218	160.9	3.6	11.5	0.2	22.2	–	2.45	2.45
CH1	205	146.9	4.7	14.9	2.2	29.3	–	2.38	2.38
CH2	203	147.4	8.1	20.6	1.7	34.1	–	2.43	2.45
GF1	223	138.4	7.5	7.6	0.4	9.2	728	2.29	2.32
GF2	213	134.2	5.4	7.9	2.4	14.3	787	2.31	2.34
PE1	138	146.5	6.5	15.5	0.9	23.7	594	2.29	2.31
PE2	110	144.4	4.4	19.1	3.3	29.4	710	2.31	2.32
CS1HS1	205	159.5	7.3	13.9	1.0	27.8	–	2.45	2.45
CS1CH1	215	156.4	9.3	23.3	1.3	33.2	–	2.45	2.45
CS1GF1	213	146.7	9.8	14.7	1.0	29.2	–	2.37	2.40
CS1PE1	100	153.7	7.8	22.6	1.2	35.0	–	2.36	2.39
GF1.5PE0.5	145	133.6	5.1	16.0	0.5	26.9	1020	2.28	2.34

The notations f^’^c and σ refer to the average compressive strength and average flexural strength

*σ represents the average of the peak flexural stresses reached during the load–deflection curve up to midspan deflection of 3.5 mm

** Mixtures with expansive behavior

### Flow

**Fig. 2:**
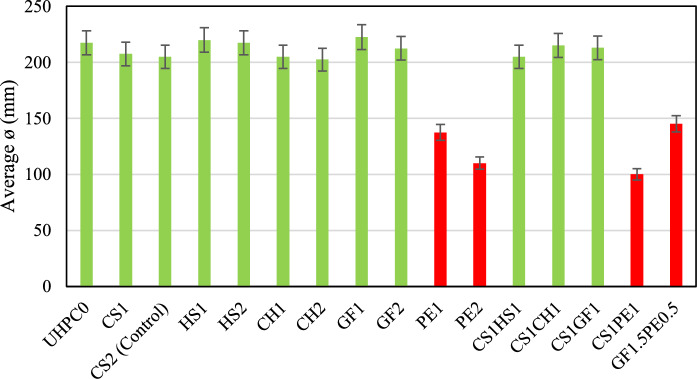
Mini slump flow diameter

To be considered a self-consolidating mixture, the UHPC should achieve a mini slump flow diameter above 200 mm. As shown in Fig. 2, all the mixture designs reached the 200 mm threshold without any adjustments in the HRWR dosage, except for those containing PE fiber. In the self-consolidating mixtures, flow varied from a decrease of 1.2% in the CH2 mixture (203 mm) to an increase of 8.5% in the GF1 mixture (223 mm) in relation to the CS2 (205 mm), the control mix. Although the mixtures with PE, CS1PE1, PE2, PE1, and GF1.5PE0.5 mixtures showed a significant decrease in the flow of 29, 33, 46, and 51%, respectively, they easily filled the molds when external vibrating energy was applied, manifesting a thixotropic behavior. This effect may be attributed to the fact that PE fibers have by far the highest aspect ratio (800) that, combined with a low modulus of elasticity, makes the filaments highly flexible, which has been demonstrated to increase the yield stress in fresh concrete and therefore reduces its flowability [15].

**Fig. 3:**
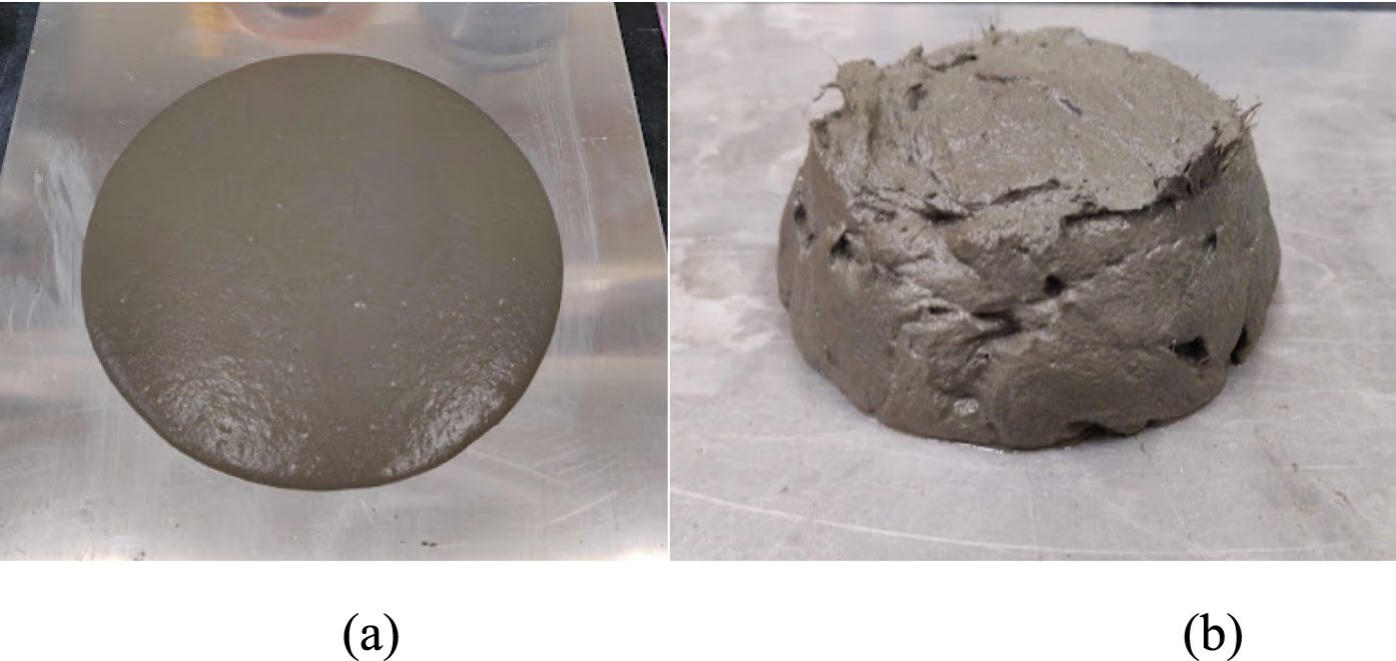
**a)** Self-compacting mixture; **b)** Mixture with PE fibers

Besides the PE fibers (Fig. 3), the types of fiber and dosages used in this study did not considerably affect the flow of fresh UHPC. Also, none of the mixtures showed fiber balling or agglomeration, which can negatively affect the hardened properties [16].

Although the group of mixes that can be classified as self-consolidating has a wide range of applications, such as precast elements, bridge joints, and structural reinforcement, among others, the thixotropic group also has some interesting potential uses such as pavement overlays and 3D printing [17].

### Density

**Fig. 4:**
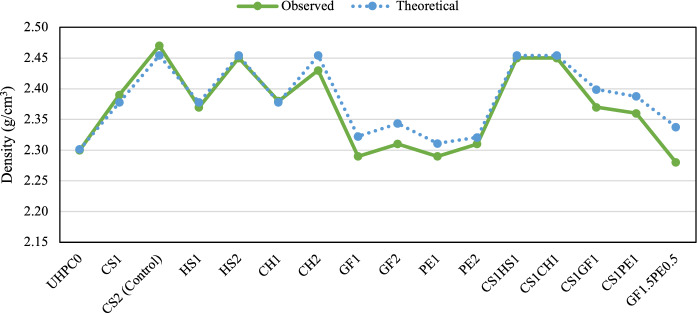
Observed and theoretical density

The control mix, CS2, had an apparent density of 2.47 g/cm^3^. Other mixes with 2% steel fibers (HS2, CS1CH1, CS1HS1, and CH2) had a negligible 0.8–1.6% decrease in the density when compared to the control; their densities were 2.45, 2.43, 2.45, and 2.45 g/cm^3^, respectively. Mixes with 2% synthetic fibers (GF2, PE2, and GF1.5PE0.5) had densities of 2.31, 2.31, and 2.28 g/cm^3^, showing a significant reduction of 6.5–7.7%. Finally, the hybrid mixes CS1GF1 and CS1PE1 had intermediate values of density of 2.37 and 2.36 g/cm^3^, a reduction of 4.0–4.5%. Even though the observed values were very close to the theoretical densities, as shown in Fig. 4, mixtures containing GF and PE fibers have a greater gap between both values, and this can be attributed to an increased porosity at the fiber-matrix interface, meaning a greater amount of entrained/entrapped air, produced by the hydrophobic nature of these fibers [18].

### Bulk resistivity

As shown in Table 5, a bulk resistivity value of 1035 Ωm was measured for the UHPC0 mixture, while mixtures with synthetic fibers such as CF1, CF2, PE1, PE2, and CF1.5PE0.5 had values between 594 and 1020 Ωm. A significant reduction in bulk resistivity with the addition of fibers was observed in some mixes, attributable to the variable moisture state of the mixes. Nevertheless, the values were still well above 250 Ωm, which suggests a negligible chloride ion penetrability, indicating a high durability [19], although the complex effect of curing on bulk resistivity complicates interpretation. The exact reasons why resistivity varies so much in these mixtures are hard to pinpoint because the degree of saturation in the mixtures was not controlled.

### Compressive strength

Results shown in Table 5, showing compressive strength, can be separated into four categories based on their performance. The first one, with CS1 and CS2 (control) mixtures, had the highest compressive strength values, 168.6 MPa, and 174.0 MPa, respectively, representing a significant increase of 15 and 19% compared to the UHPC0 mixture that had no fibers, primarily due to the confinement effect of the carbon steel microfibers [20]. In the second category, CS1CH1, CS1HS1, and HS2 mixtures showed a slight compressive strength detriment between 8 and 10% compared to the control mixture (CS2). This reduction may be attributed to the reduced confinement effect, which is less pronounced in these mixtures due to the hooked-end fiber geometry and larger fiber lengths. Hung et al. reported that crack size is proportional to fiber scale, meaning that replacing micro-fibers with macro-fibers led to larger crack formations and a higher probability of defects at the fiber-matrix interface [21]. Additionally, the reduced compressive strength could also be partially attributed to increased air entrapment during the mixing process. Yu et al. demonstrated that air content and porosity in UHPC increase parabolically with higher fiber content [22]. HS1, CH1, CH2, PE1, PE2, CS1GF1, and CS1PE1 form the third category and showed a decrease in compressive strength between 11 and 17% compared to CS2, a similar behavior to the UHPC0 which suggests that the fibers had virtually no effect on the compressive strength. Finally, GF1, GF2, and GF1.5PE0.5 exhibited a detriment of the compressive strength between 20 and 23%, compared to CS2. The relatively poor compressive performance of certain mixtures can be attributed to a combination of a reduction of the confinement effect and air entrapment at the fiber-matrix interface.

### Flexural performance

**Fig. 5:**
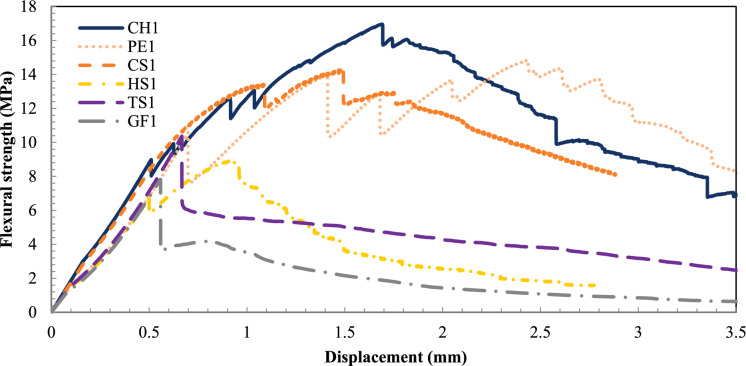
Flexural strength vs. deflection curves for 1% addition of fiber

**Fig. 6:**
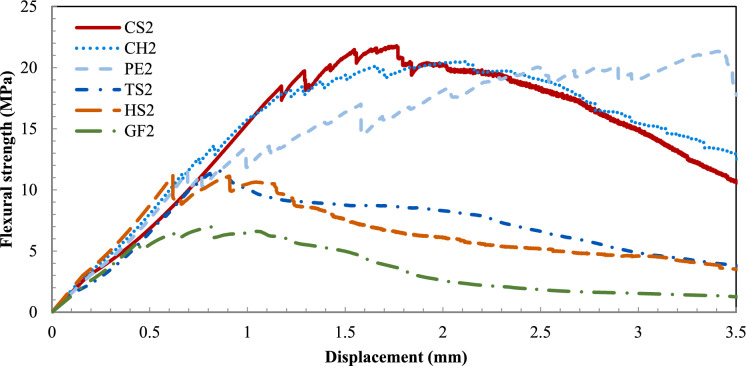
Flexural strength vs. deflection curves for 2% addition of fiber

**Fig. 7:**
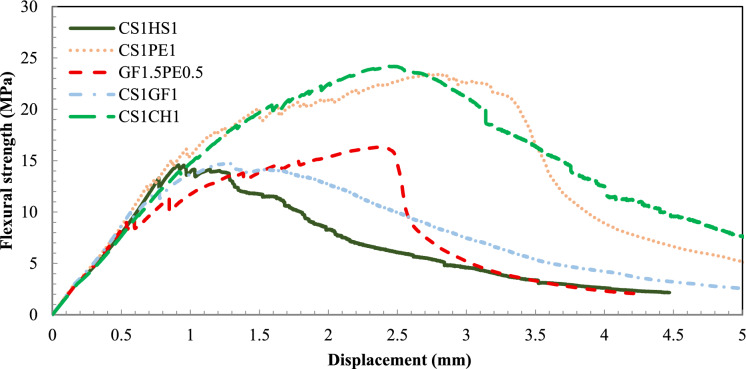
Flexural strength vs. deflection curves for 2% addition of fiber combinations

Figures 5, 6, and 7 show representative flexural strength vs deflection curves for each mixture, highlighting post-cracking behavior and residual strength trends. While the results discussed refer to the average values from Table 5, the curves effectively reflect characteristic differences among fiber types and dosages. In evaluating the mixtures flexural performance, a baseline was established with the UHPC0, which had no fibers. The flexural strength of UHPC0 was 10.9 MPa on average. At 1% addition of fiber, the PE1, CH1, and CS1 mixtures had the highest average flexural performances, achieving 15.5, 14.9, and 14.5 MPa, respectively, while HS1 (8.1 MPa) and GF1 (7.9 MPa) did not even achieve the UHPC0 threshold. Figure 5 shows where the post-cracking hardening effect was evident in the PE1, CH1, and CS1 mixtures. It also shows the much higher strain capacity of the PE1 mixture, where the peak load was achieved around the 2 mm midspan deflection.

At 2% addition of fibers (Fig. 6), the PE2, CH2, and CS2 mixtures still had the highest flexural performance with 19.1, 20.6, and 20.8 MPa, displaying a consistent increase, between 26 and 30%, of the flexural strength compared to their corresponding 1% mixes. Therefore, CH and PE appear to be the most promising to potentially replace the CS fibers. The HS2 mixture achieved an average flexural strength of 11.5 MPa, barely surpassing the UHPC0 threshold, while GF2 did not significantly change its performance. This is in contrast with earlier findings [22–24]. Deformed fibers can demonstrate superior performance in enhancing the tensile and flexural properties of UHPC due to the additional resistance provided by mechanical anchorage and interlocking. On the other hand, HS fibers have a diameter of 2.75 times that of CS fibers. Their greater stiffness likely caused crushing of the concrete matrix during flexural loading, resulting in premature failure and reduced toughness. Qiu et al. highlighted that excessively reducing the aspect ratio by using overly large fiber diameters can limit ductility and accelerate crack propagation during the early softening phase [25]. Similarly, it has been observed that increasing the dosage of GF beyond 0.5% does not result in a significant improvement in flexural strength [26]. Also, GF, due to their brittle nature, are prone to fracture under high stress, which limits their ability to enhance toughness and post-cracking ductility. A very similar behavior between CS2 and CH2 can be observed in Fig. 6, where the peak load is achieved close to the 1.5 mm midspan displacement, and the material softening part of the curves overlap. It is also evident that the PE2 mix has a high strain capacity, achieving its peak load around the 3 mm midspan displacement.

For Phase 2, five combinations of fibers, with a total fiber content of 2% were studied as detailed in Table 4. As shown in Fig. 7, CS1CH1 and CS1PE1 mixes had the highest average flexural strength, with 23.3 and 22.6 MPa, respectively. These two results reflect a synergistic behavior since the combined performance is between 9 and 18% higher than that of the single fiber mixes at 2%. CS1GF1 (14.7 MPa) and CS1HS1 (13.9 MPa) had a similar performance to CS1 (14.5 MPa), indicating that neither the GF nor the HS fibers had any significant impact on the flexural strength. The only non-metallic fiber combination, the GF1.5PE0.5, had an average flexural strength of 16 MPa, comparable with CS1, CH1, and PE1 performances, and was thus not a promising outcome.

Overall, the standard deviation (SD) of the flexural strength results ranged from 0.2 to 3.3 MPa. Higher SD values were generally observed in mixtures with long or flexible fibers (e.g., PE and CH), where random orientation effects are more pronounced. This level of scatter is consistent with ranges typically reported for UHPC systems [16]. Such variation is expected due to the sensitivity of fiber-reinforced systems to factors like fiber alignment, mixing uniformity, and localized bridging.

**Fig. 8:**
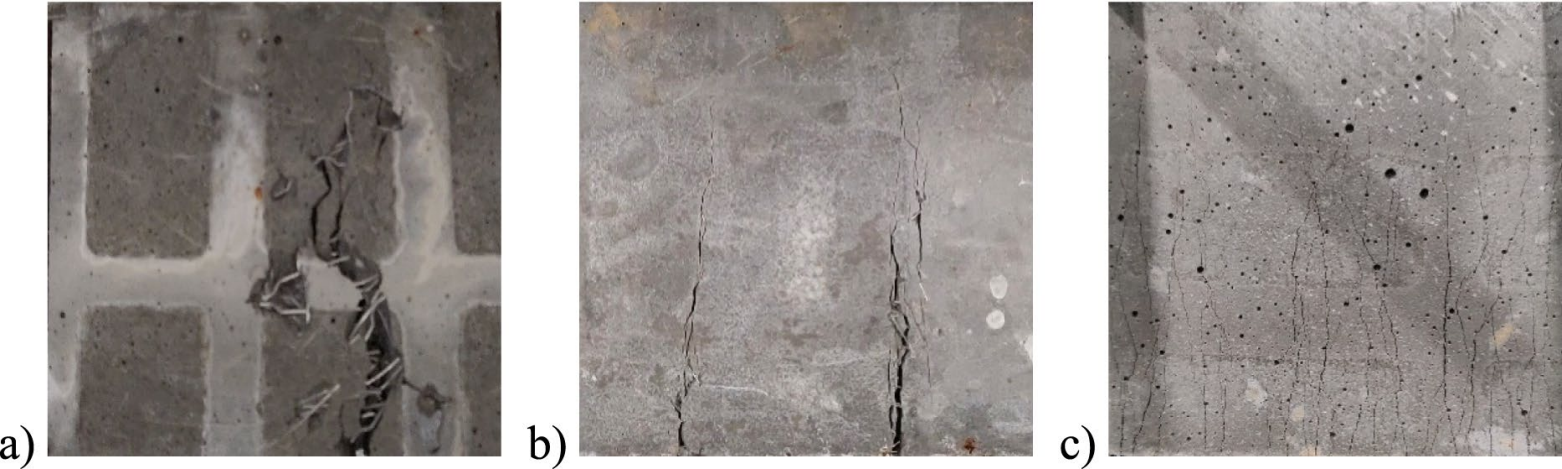
Cracking patterns: **a)** localized single crack; **b)** multiple cracking; **c)** highly distributed multiple cracking

The T150 toughness is represented by the area under the load–displacement curve up to a midspan deflection equal to the total beam span over 150 (L/150), in this case, 1.7 mm. This value determines the energy absorption capacity of the material through deformation. For Phase 1, CS2, CH2, and PE2 exhibited the highest T150 values of 34.7, 34.1, and 29.4 J, respectively. The second phase showed that CS1PE1 and CS1CH1 had higher toughness values of 35 and 33.2 J. The different observed cracking propagation patterns seem to be related to the toughness capacity. GF1, HS1, and GF2, the mixtures with lower T150 toughness, had localized single crack failure patterns, as shown in Fig. 8a, while the rest of the mixtures, with higher T150 toughness, exhibited multiple cracking patterns, such as the ones pictured in Fig. 8b and c.

**Fig. 9:**
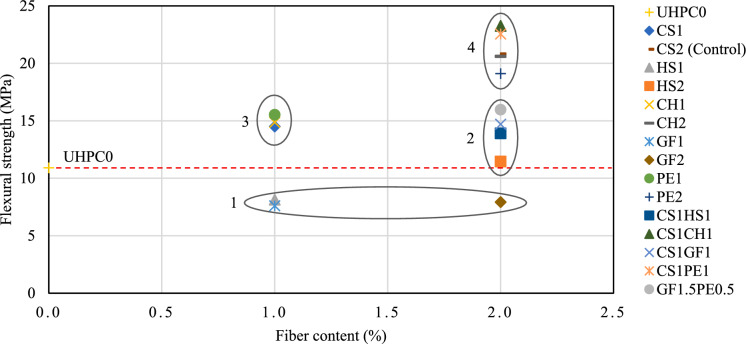
Mixture classification by performance and fiber content

Plotting the average flexural strength vs. the fiber content allows the classification of the different mixtures into different performance groups, as shown in Fig. 9. Group 1 is composed of mixtures that did not surpass the UHPC0 threshold, GF1, HS1, and GF2. Group 2 contains the mixtures with intermediate performance with 2% fiber content, HS2, CS1HS1, CS1GF1, and GF1.5PE0.5. Group 3 are mixtures with intermediate performance but with only 1% fiber content, CH1, CS1, and PE1. Finally, Group 4 comprises the higher performance mixtures at 2% fiber content, PE2, CH2, CS2, CS1PE1, and CS1CH1. Mixtures in groups 3 and 4 are preferred for their performance.

**Fig. 10:**
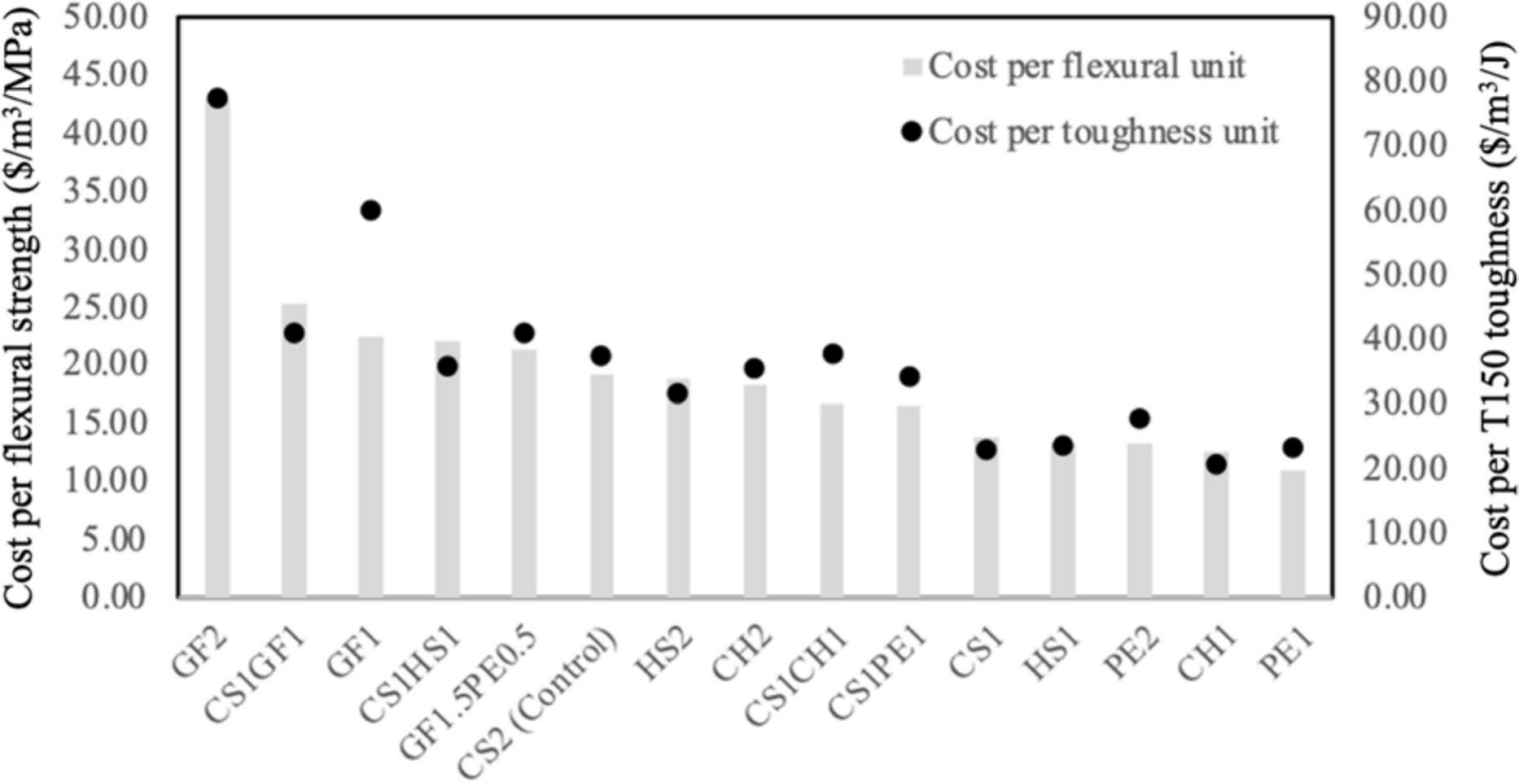
Cost per flexural strength and toughness unit

The unitary flexural strength cost was calculated and graphed to assess the performance of the fibers from an economic aspect, as shown in Fig. 10.

The mixtures have a higher cost per cubic meter per flexural strength unit (MPa) from left to right. This means that mixtures located to the left of CS2, the control mix, have higher unitary flexural strength costs than the control UHPC, and they also generally happen to be the mixes with the lowest performance corresponding to groups 1 and 2. On the other hand, the mixtures located to the right of CS2, all from groups 3 and 4 except for HS1 and HS2, have lower unitary flexural strength costs than the control UHPC. All the prices were obtained directly from the fiber producers, and all fibers were produced in the US. CS1CH1 and CS1PE1 can be highlighted here, as they achieve the best flexural performance while reducing units costs by 13% and 14.5% respectively, when compared to CS2.

PE1, PE2, and CS1PE1 are extremely promising from flexural strength and cost perspectives. However, these mixtures showed relatively poor flow and compressive strength, or at least reductions when compared to control. GF and TS did not show promising behavior. Many of the mixtures with other steel fibers, as an example, CS1CH1 showed behavior comparable to CS2; in principle CS1CH1 could replace CS2 with similar flowability, strength, and toughness, while offering some cost and flexural advantages. Clearly, there are complex cost, compressive strength, flexural strength, toughness, and flow tradeoffs, as there are also synergies. We do not delve into the optimization of these fibers and mixtures, but plots such as Fig. 10, using compressive strength, flexural strength, and flow normalized to cost, help in identify fiber combinations that provide the best performance for the required application.

## Conclusions

Supported by the results of this study, the following conclusions were drawn:

- The studied fiber types and dosages generally had little effect on mixture flowability, with most showing variations of − 1.2 to + 8.5% relative to the control (CS2), indicating that all mixtures maintained self-consolidating behavior. However, mixtures containing PE fibers exhibited a substantial reduction in flowability, between 29 and 51%, due to increased yield stress associated with filament flexibility, high aspect ratio, and low modulus of elasticity. Mixtures with limited flowability may be suitable for applications such as 3D printing or pavement overlays. However, further research is needed to optimize their rheology for broader use requiring adequate flow properties.
- The UHPC matrix exhibited a very high bulk resistivity (1035 Ωm), indicating extremely low permeability and excellent durability. Although the inclusion of non-metallic fibers reduced bulk resistivity in some cases (594–1020 Ωm), the values remained well above the thresholds expected for negligible chloride ion penetration, confirming that durability was unlikely to be compromised by fiber addition.
- Fibers significantly influenced the compressive strength of UHPC, primarily through their ability to confine the matrix during loading, which depends on their material properties, size, and geometry. Mixtures with CS fibers (CS2 and CS1) achieved compressive strength increases of 19% and 17%, respectively, compared to UHPC0. In contrast, GF fibers were less effective, with the GF2 mixture showing up to an 8.3% reduction in compressive strength, attributed to additional air entrapment confirmed by density measurements.
- The flexural strength evaluation in Phase 1 (single fiber type) indicated that CH and PE fibers are the most promising alternatives for partially or fully replacing CS fibers. At 1% and 2% dosages, both fibers achieved performance comparable to CS, with flexural strengths of 14.9 and 15.5 MPa for CH1 and PE1, and 20.6 and 19.1 MPa for CH2 and PE2, respectively.
- In Phase 2 (binary fiber combinations), a synergistic effect was observed in the flexural performance of CS1CH1 and CS1PE1 mixtures. Compared to their corresponding single-fiber mixtures at the same total fiber content (2%), these combinations achieved flexural strength increases of 9 to 18%.
- The unitary flexural cost showed that mixtures in groups 3 and 4 were the most economical. Among them, CS1CH1 and CS1PE1 achieved the highest flexural performance, exhibiting unit costs per MPa that were 13% and 14.5% lower, respectively, than the control mixture (CS2). These results suggest potential for further optimizing such mixtures, reducing the fiber dosage to match the control’s flexural performance, and potentially reducing the overall costs even further.
- A hybrid mixture containing 1% CS fibers and 1% CH fibers (CS1CH1) can effectively replace the control mixture with 2% CS fibers (CS2), offering comparable flowability, compressive strength, and T150 toughness, while delivering superior flexural performance and cost efficiency.

## Data Availability

Data will be made available upon reasonable request.
